# Genetic overlap between major depressive disorder and obstructive sleep apnea

**DOI:** 10.3389/fpsyt.2024.1464396

**Published:** 2024-11-05

**Authors:** Fangbo Lin, Yanyao Deng, Le Xiao, Chao Liu, Jie Li

**Affiliations:** Rehabilitation Medicine Department, The Affiliated Changsha Hospital of Xiangya School of Medicine, Central South University (The First Hospital of Changsha), Changsha, China

**Keywords:** major depressive disorder, obstructive sleep apnea, GWAS, genetic overlap, genetic correlation

## Abstract

**Objective:**

Observational studies have frequently shown a co-occurrence of psychiatric disorders and Obstructive sleep apnea (OSA), with major depressive disorder (MDD) being a prevalent psychiatric disorder. This study aims to investigate the genetic overlap between MDD and OSA to explore their underlying pathological mechanisms.

**Methods:**

Leveraging the extensive and recent GWAS for OSA and MDD, we conducted genetic correlation analyses utilizing Linkage disequilibrium score regression (LDSC), re-evaluated their pleiotropic Single-nucleotide polymorphisms (SNP) with Cross-Phenotype Association (CPASSOC) and Colocalization (COLOC), investigated the overlap at the gene level using physical annotations and Multi-marker Analysis of GenoMic Annotation (MAGMA), and finally employed Mendelian randomization (MR) to assess potential causal relationships between the two disorders.

**Results:**

Upon our investigation, we established that MDD and OSA exhibit high heritability (h2MDD=0.02, h2OSA=0.04) alongside a significant genetic correlation (rg=0.31, P= 1.42E-23). Utilizing CPASSOC, we identified 397 pleiotropic SNPs, associable with 45 loci, two of which share common genetic fragments with a pleiotropic role. Furthermore, the MAGMA study uncovered a total of 154 pleiotropic genes capable of influencing multiple brain regions. Lastly, leveraging MR analysis, we concluded that MDD heightens the risk of developing OSA (P=3. 10E-04, OR (95%CI):1.28(1.12~ 1.47)).

**Conclusion:**

In summary, our study identified *PCLO* as a common gene between OSA and MDD and provided evidence that MDD causally contributes to the development of OSA. These insights enhance our understanding of the shared mechanisms underlying the comorbidity of these conditions.

## 1 Introduction

Obstructive sleep apnea (OSA) is a common sleep disorder characterized by apnea and hypoventilation due to narrowing of the airway during sleep. Because of intermittent hypoxia and sleep fragmentation, OSA is often associated with a series of metabolic disorders and multiple system injuries (1). OSA and various psychiatric disorders often present with similar symptoms, such as fatigue, lethargy or insomnia. Mental disorders, along with obesity and metabolic dysfunction, are independent predictors of OSA (2). Major depressive disorder (MDD) is a common and highly heterogeneous psychiatric disorder characterized by chronic low mood.

The risk of comorbid OSA with MDD and the risk of comorbid MDD with OSA are both much higher than in the general population. Obesity, dyslipidemia, diabetes, and other metabolic dysfunctions are prevalent in patients with OSA and MDD (3). However, the underdiagnosis and untreated rates of OSA in MDD patients are also very high (4, 5). OSA in psychiatric patients is likely to exacerbate the severity of their mental illness if not diagnosed and treated in a timely manner (6). Psychiatric disorders, on the other hand, can have a serious impact on treatment adherence and quality of life for patients with OSA (7). According to surveys, the prevalence of OSA in men and women was 3-7.5% and 2-3% respectively, however, the prevalence of OSA in psychiatric patients can be as high as 15-48% (8). Therefore, it is necessary to explore the potential mechanisms underlying the correlation between OSA and MDD. There is a genetic role in the etiology of both OSA and MDD, and the aim of this study was to explore whether both are determined by the same portion of genetic variation. The link between depression and OSA may be closely related to the dysregulation of serotonin and dopamine levels (9), as well as the effects of intermittent hypoxia. The polymorphism rs2770304 in the serotonin receptor encoding gene *HTR2A* increases the risk of developing the condition (10). Conversely, research shows that in OSA patients, chronic intermittent hypoxia increases the expression of monoamine oxidase A (MAO-A), which accelerates the breakdown of serotonin, leading to lower serotonin levels. Xiong found that intermittent hypoxia exacerbated depressive and anxiety-like behaviors in bleomycin-induced pulmonary fibrosis mice (11).Additionally, intermittent hypoxia may cause dysregulation in dopamine regulation in the brain, further aggravating emotional and cognitive dysfunctions and worsening depressive symptoms (12). Therefore, depressive symptoms in OSA patients may arise from the imbalance of serotonin and dopamine, as well as the neurodegenerative effects caused by hypoxia.

Previous studies have demonstrated that genetic factors play a significant role in the development of both OSA and MDD. Genome-wide association studies (GWAS) are a method for identifying genetic regions associated with specific phenotypes or diseases. This approach involves detecting hundreds of thousands to millions of genetic variants in the genome that exhibit significant associations with these traits or conditions (13). Quinlan et al. conducted a GWAS on OSA in European American and African American children, identifying genetic loci associated with age-related OSA (14). Meanwhile, Shi et al. conducted a GWAS in a Han Chinese population, identifying novel genetic loci associated with OSA, which suggest that *SLC52A3* may serve as a key therapeutic target (15). Xu et al. utilized Mendelian randomization (MR) methods based on GWAS to investigate the causal relationship between depression and delirium (16). Traditional genetic research typically focuses on the effects of genetic variations on a single disease or trait. However, research on genetic overlap has revealed shared genetic variations between different diseases, providing insights into deeper pathological mechanisms. This approach complements the limitations of clinical studies by offering a more comprehensive understanding of the genetic factors underlying complex disorders.

By integrating data from large-scale GWAS, we not only analyze the genetic association between MDD and OSA but also explore the genetic overlap between them at the Single Nucleotide Polymorphism (SNP) and gene levels. We performed linkage disequilibrium score regression (LDSC) to estimat the genetic correlation between OSA and MDD. Additionally, we pinpoint specific pleiotropic genomic regions and pathways enriched in both disorders. Colocalization analysis (COLOC) was conducted to determine whether shared loci indeed reflect shared causal variants or are simply in close proximity. Finally, we used MR to analyze whether there is a causal component between the two traits, and enrichment analysis to determine the underlying pathophysiological mechanisms. In general, we conducted a cross-trait analysis using GWAS of MDD and OSA to investigate the particular genetic mechanisms involved, thus offering theoretical support for future research and management of sleep disorders in psychiatric patients.

## 2 Methods

**Figure 1:**
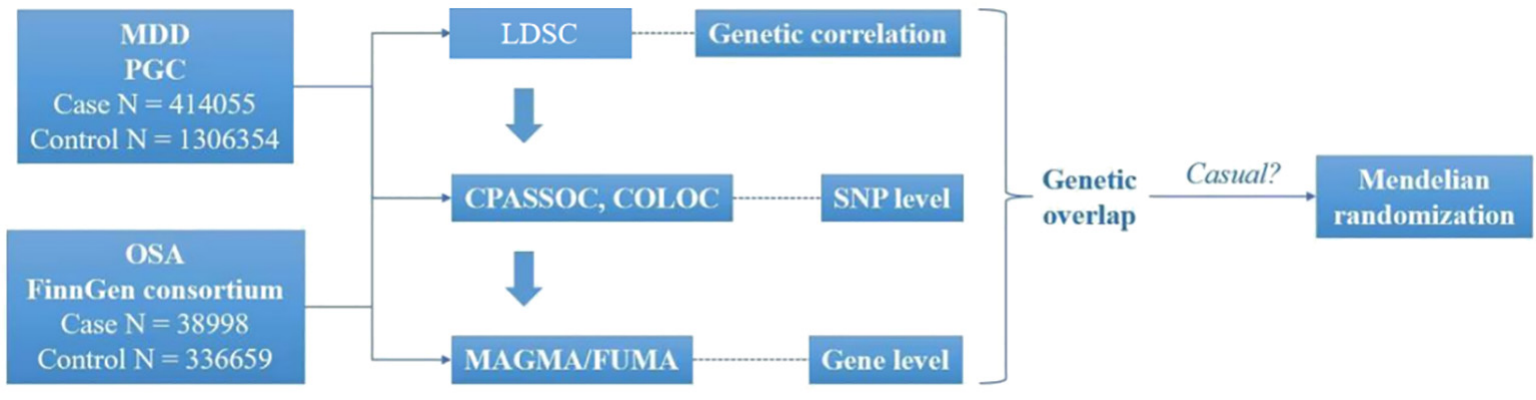
Overview of research of shared genetic architecture between MDD and OSA. COLOC, Colocalization; CPASSOC, Cross- Phenotype Association; FUMA, Functional mapping and annotation; LDSC, Linkage disequilibrium score regression; MAGMA, Multi-marker Analysis of GenoMic Annotation; MDD, Major depressive disorder; OSA, Obstructive sleep apnea. SNP, Single-nucleotide polymorphisms.

The GWAS for MDD were obtained from the Psychiatric Genomics Consortium (https://pgc.unc.edu/), comprising 414055 cases and 1306354 controls. Summary GWAS for OSA comprising 38998 cases and 336659 controls, were obtained from FinnGen consortium R9 (https://www.finngen.fi/en/access_results). The 1000 Genomes European population were served as the reference. We standardized both GWAS datasets by imputing missing data for Chromosome (CHR), Base Pair (BP), effect allele (A1), reference allele (A2), beta, standard error (SE), and *P*-values. We also eliminated overlapping entries, excluded “NA” values, and ensured consistent gene orientation across datasets. Furthermore, the selected databases met the fundamental criteria for Mendelian randomization (MR), specifically using data from different populations within the same ethnic group (**Figure 1**).

### 2.1 Linkage disequilibrium score regression

We conducted a LDSC analysis to estimate the heritability of each condition (single-trait) and evaluate the genetic correlation between OSA and MDD (ranging from -1 to 1) (17). It evaluates the relationship between LD scores of SNPs across the genome and GWAS z-scores to account for potential confounding effects arising from linkage disequilibrium. This approach allows for more accurate estimation of heritability and the genetic correlation between traits. Phase 3 of the 1000 Genomes project (18) of Europearn population provide the precomputed linkage disequilibrium (LD).

### 2.2 SNP level analysis

After calculating the genetic relationship between MDD and OSA, we applied the Cross-Phenotype Association (CPASSOC) algorithm (19), which integrates GWAS data from multiple traits to reduce heterogeneity and enhance statistical power. CPASSOC offers two distinct statistical approaches: SHom and SHet. The SHom model employs a fixed-effects meta-analysis approach, which is particularly effective for detecting homogeneous genetic effect sizes. In contrast, the SHet model extends SHom by increasing sensitivity to heterogeneous effects that may arise from differences in study designs, environmental influences, or populations. Significant SNPs were defined by thresholds of P<1E-3 for single-trait analysis and P<5E-8 for CPASSOC analysis. Novel SNPs were identified with 0.001 < P < 5E-8 in single-trait analysis and P<5E-8 in the meta-analysis. Polytomous analyses centered on meta-algorithms may only give us results that are associated with only one trait, so we used COLOC (20),which utilizes Bayesian algorithms, to determine whether two different traits share the same genetic information in a given region. The method generates posterior probabilities for five mutually exclusive hypotheses based on two traits, including H0 (no association), H1 or H2 (associated with only one trait), H3 (associated with two traits with different genetic information in the same genetic segment), and H4 (associated with two traits with the same genetic information in the same genetic segment). One limitation of GWAS is that, although it can identify genetic loci associated with specific traits, many variants reside in non-coding regions, and LD spreads statistical signals across multiple loci. Therefore, further analyses at the genetic level are essential to pinpoint the causal variants and understand their functional impact. After discovering pleiotropic SNPs, we used functional mapping and annotation (FUMA) (21) to locate independent genomic loci with information on the SNP’s functional class, CADD score, RegulomeDB score, and chromatin status. Within each locus, The SNP with the lowest P-value was designated as the top SNP. Independent and significant SNPs were identified based on their linkage disequilibrium (LD), measured by the r^2^ value, in relation to the top SNP. We extracted summary statistics for variants within 500 kb of the topSNP on each shared locus and calculated the posterior probabilities for H4 (PPH4) and H3 (PPH3). A locus was considered co-localized if PPH4 or PPH3 was greater than 0.7.

### 2.3 Genetic level analysis

Genome analyses are considered to be potentially more powerful alternatives to the typical single SNP analyses performed in GWAS. The SNPs located in the same or nearby region are physically annotate into genes by FUMA. In addition to integrating genes with complex trait information, we used Multi-marker Analysis of GenoMic Annotation (MAGMA) (22), a flexible sensitive method utilizing a multiple regression approach to properly incorporate LD between markers to detect multi-marker effects for traits. During the analysis, genes located within 50 kb of each candidate SNP were mapped and prioritized based on their linkage disequilibrium (LD) with genome-wide significant SNPs, determined by the adjusted \(r^2\) threshold. Subsequently, we conducted an overlap analysis between the gene sets derived from MAGMA and those identified by FUMA, considering overlapping genes as potential pleiotropic candidates.

### 2.4 Enrichment

To explore the anatomical organization and functional changes of these gene dependencies, we further performed tissue enrichment analysis and Gene Ontology (GO) analysis (23) using the gene function module on the FUMA platform to identify the enrichment of pleiotropic genes. The GTEx version 8 reference panel was applied for tissue enrichment analysis, and results were adjusted for multiple comparisons using the False Discovery Rate (FDR) (24) and Bonferroni correction (25), respectively.

### 2.5 Mendelian randomization

Based on the previous methods we can get whether OSA and MDD were genetically correlated, next we estimated whether genetic factors played a causal role, MR (26), an algorithm that uses genetic variation as an instrumental variables (IV) to estimate the causality between the relevant exposure factor and the relevant outcome. The instrumental variables (IVs) must be strongly associated with the exposure and independent of the outcome, typically defined by a threshold of P< 5E-8. Additionally, it is essential to account for horizontal pleiotropy and ensure the strength of the IVs, with an F-statistic > 10 considered adequate. The inverse variance weighting (IVW) method served as our primary approach for MR analysis, while the MR-Egger and weighted median methods were employed to assess the robustness of the results under more relaxed model assumptions. To assess the stability and reliability of the results, we conducted gene multiplicity and heterogeneity tests. Additionally, we applied the “leave-one-out” method, sequentially removing each SNP to calculate the meta-effects of the remaining SNPs. This approach allowed us to determine whether the exclusion of specific SNPs would lead to significant changes in the overall results.

## 3 Results

### 3.1 LDSC

Genome-wide genetic correlation quantifies the extent to which two traits share genetic influences, independent of environmental confounders. Using LDSC, the heritability estimates were 0.04 for OSA and 0.02 for MDD. The genetic correlation analysis (rg) revealed a significant genetic correlation between MDD and OSA (rg = 0.31, P = 1.42E-23).

### 3.2 CPASSOC and COLOC

Through CPASSOC analysis, we identified 397 pleiotropic SNPs (**Supplementary Table 1**), These SNPs exhibited effects on traits were almost uniformly in the same direction, including 239 newly discovered pleiotropic SNPs (**Supplementary Table 2**), The most significant SNP identified in this study was rs2043539, which located at CHR: BP = 7:12253880 (PCPASSOC= 1.64E-13). These SNPs were mapped to a total of 45 loci based on their physical locations. Among these, COLOC analysis identified two loci with PPH4 values greater than 70%, suggesting the presence of genetic fragments within these loci that may have pleiotropic effects, influencing multiple traits or biological processes. The topSNPs corresponding to these loci were rs1228509 (PPH4 = 0.82) and rs59333125 (PPH4 = 0.79), mapping to the *EPHB2* and *PCLO* genes, respectively (**Table 1**).

**Table 1:** Significant results from colocalization analysis for each pleiotropic locus, CHR: Chromosome, BP: Base Pair, MDD: major depressive disorder, OSA: Obstructive sleep apnea, PPH4: posterior probabilities for H4, PPH3:posterior probabilities for H3.

TopSNP	CHR	BP	Locus	MDD		OSA		PCPASSOC	PHH3	PHH4	Genes
				Beta	*P*-value	Beta	*P*-value				
rs1228509 rs59333125	1 12	97030144 57025908	2 30	0.009 -0.004	0.04 0.6	-0.047 -0.082	6.41E-09 1.32E-08	1.71E-08 3.57E-08	0.06 0.09	0.82 0.81	EPHB2 PCLO

### 3.3 MAGMA

**Figure 2:**
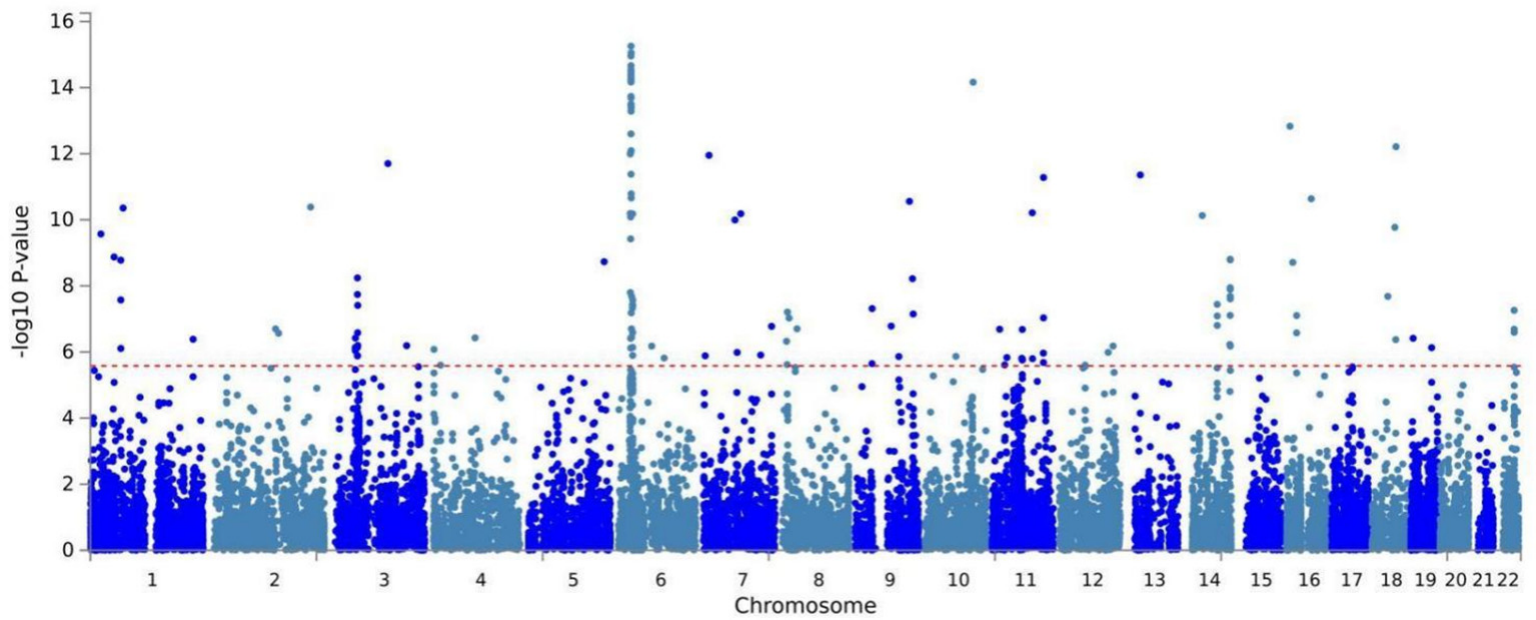
Manhattan plot of the shared genes of OSA and MDD by MAGMA identified from CPASSOC.

Building on physical annotation, we identified an additional 229 genes through the MAGMA method (**Supplementary Table 3**). After applying Bonferroni correction, 154 genes remained significant (**Supplementary Table 4**). The MAGMA analysis results for genomic risk loci mapped to genes meeting the COLOC criteria (PPH4 > 70%) were also significant, Specifically, *EPHB2* exhibited a P_MAGMA_ = 2.69E-10 and *PCLO* had P_MAGMA_ of 6.55E-11 for. The Manhattan plot for pleiotropic genes is shown in **Figure 2**.

### 3.4 Enrichment

**Figure 3:**
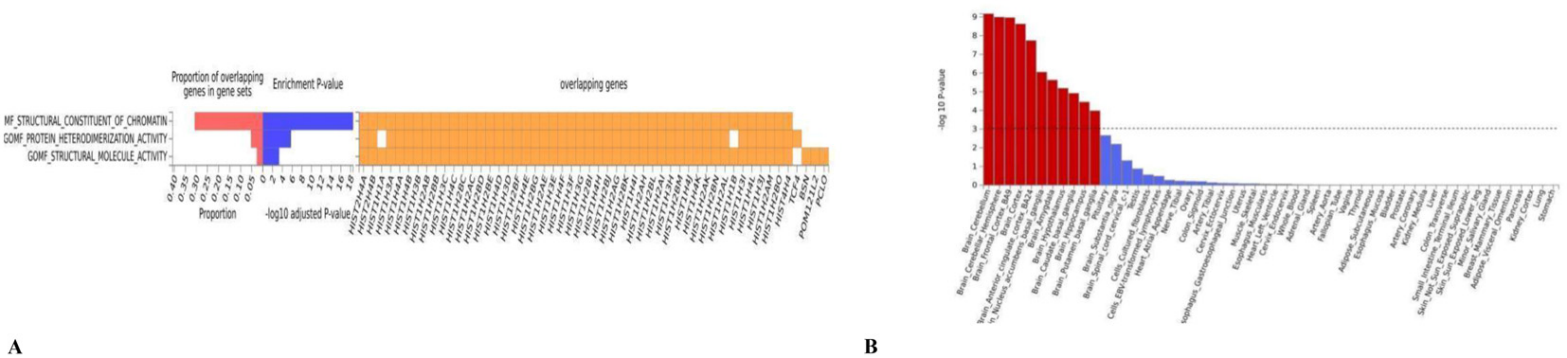
**(A)** The GO result of the pleiotropic genes; **(B)** the result of tissue enrichment in GTEx 8 on the pleiotropic genes, the color red represents the significant tissue, the color blue represents the tissue did not reach threshold.

In the functional enrichment, the FDR-corrected gene dependencies were primarily associated with structural molecular activity (P= 1.00E-06) and protein heterodimerization activity (P=3.00E-02) (**Figure 3A**). In tissue enrichment analysis, we found that the genetic overlap of MDD and OSA can affect the co-occurrence by regulating 13 brain regions and pituitary gland (**Figure 3B**, **Supplementary Table 5**). The most significant brain region identified was the cerebellum (P = 6.75E-10), followed by the cerebellar hemisphere (P = 1.02E-09).

### 3.5 MR

After screening a total of 40 instrumental variables, all of which exhibited strong instrumental characteristics, with the lowest F value being 215 (**Supplementary Table 6**), we observed that MDD contributed to an increased incidence of OSA through the IVM (P=3. 10E-04, OR(95%CI): 1.28(1. 12~ 1.47)) and Weighted Median methods (P=6.72E-03, OR(95%CI): 1.22(1.06~ 1.42)). The results obtained using other methods were consistent with this findings (**Table 2**). Heterogeneity analysis revealed a P_het_ value of 3. 10E-04, significantly below 0.05, which justified the application of a random effects model. In our comprehensive multivariate analysis, the obtained P value was notably greater than 0.05 (P = 0.85), and the “leave-one-out” analysis demonstrated that excluding each SNP did not alter the direction of the results, confirming the robustness of our findings.

**Table 2:** MR results of causal effects between MDD and OSA.

Exposure	Outcome	Method	nsnp	b	se	pval	OR(95%CI)
MDD	OSA	MR Egger	40	0.17	0.44	7.02×10- 1	1. 18(0.50 to 2.79)
MDD	OSA	Weighted median Inverse	40	0.2	0.07	6.72×10-3	1.22(1.06 to 1.42)
MDD	OSA	variance weighted	40	0.25	0.07	3. 10×10-4	1.28(1. 12 to 1.47)
MDD	OSA	Simple mode	40	0.19	0.19	3.30×10- 1	1.21(0.83 to 1.77)

CI, confidence interval; nSNP, n single nucleotide polymorphism; OR, Odds ratio; MDD, Major depressive disorder; OSA, Obstructive sleep apnea.

## 4 Discussion

The present study confirmed a genetic relationship between OSA and MDD, consistent with previous findings. Additionally, this study identified the key gene *PCLO* within the overlapping genetic structure and demonstrated that MDD is the cause affecting the occurrence of OSA by MR.

Previous studies have found an association between variant rs2522833 of the Piccolo-encoding gene PCLO and MDD (27). The *PCLO* gene encodes a presynaptic cytomatrix protein that influences the release of monoamine neurotransmitters, which modulate the hypothalamic-pituitary-adrenocortical (HPA) system (28). A study investigating the effect of *PCLO* on antidepressant treatment efficacy in MDD patients found statistically significant differences in the combined dexamethasone/corticotropin-releasing hormone test results between patients with different genotypes at admission and after 4 weeks of treatment. The authors suggested that the effect of *PCLO* on hypothalamic monoamine neurotransmitter release may play a roal in regulating of the HPA axis during antidepressant treatment in MDD patients (29). Thus, our study may suggests that MDD and OSA may be jointly influenced by alterations in the HPA axis mediated by *PCLO*. Both OSA and MDD are thought to be associated with HPA axis dysregulation. It has been proposed that hypoxia and sleep fragmentation due to OSA activates the stress system including the sympathetic and HPA axes, thereby contributing the development of their comorbidities (30),

Additionally, obesity-related metabolic dysregulation, inflammation and HPA axis dysfunction in MDD may provide a potential link between MDD and various somatic diseases (31). Chronic inflammation caused by obesity inhibits the effects of leptin on the central nervous system, and leptin is not only an anti-obesity hormone, but also possesses an antidepressant effect (32). The decreased activity and unhealthy diet associated with MDD can further promote an inflammatory state, creating a vicious cycle detrimental to both physical and mental health. Chronic stress from MDD is perceived via the cerebral cortex and then transmitted to the hypothalamus to activate the HPA axis (33), which results in a series of metabolic dysregulations such as increased cortisol (COR) release and glucocorticoid resistance (34). A meta-analysis of HPA axes in older adults with MDD showed that those with MDD had higher basal COR levels at any time of the day compared to healthy controls (35). Nighttime fluctuations in COR at night are closely related to sleep and may be an underlying mechanism for various sleep disorders. Research on how OSA affects the HPA axis has yielded inconsistent results. Karaca et al. found that serum basal COR levels, and COR response levels to stimulation tests assessing the HPA axis were lower in OSA patients than in controls (36). Zhang et al. observed that the number of MDD patients with OSA who had elevated COR was higher than that of patients without OSA, They also found that adrenocorticotropic hormone levels in the combined OSA group were correlated with reduced slow-wave sleep, suggesting hyperactivity of the HPA axis in MDD patients with OSA (37). A study examining adrenal volume in OSA patients found that those with severe OSA had larger adrenal volumes than those with moderate or mild OSA, and that a higher arousal index was a significant determinant of increased adrenal volume. The authors concluded that OSA is involved in the hyperactivation of the HPA axis, reflecting the cumulative effect of OSA on this system (38).

This study has several strengths. First, it is the first study to use large-scale GWAS data to identify a common genetic structure between OSA and MDD. Second, the use of LDSC allowed us to explore genetic correlations while reducing genetic correlation variance. Finally, we employed CPASSOC to gain deeper insights into the genetic structure shared by OSA and MDD. CPASSOC considers traits, demographics, latent associations, and sample overlap.

However, this study also has limitations. We did not utilize GWAS data from other ethnicities, and the sample size for OSA was relatively small. Nevertheless, we strictly utilized GWAS data from the same ethnic group across different databases in our genetic correlation analysis to minimize the risk of sample overlap. In the future, as larger and more diverse GWAS datasets become available, we can further refine our analysis of genetic architectures at the transcriptomic and proteomic levels.

## 5 Conclusion

In conclusion, we identified *PCLO* as a gene shared by OSA and MDD, and demonstrated that MDD is a causal factor influencing the occurrence of OSA. These findings will contribute to a better understanding of the comorbid mechanisms of OSA and MDD.

## Data Availability

The original contributions presented in the study are included in the article/**Supplementary Material**. Further inquiries can be directed to the corresponding author.
